# A Plea for the Private Nursing Home

**Published:** 1912-08-10

**Authors:** 


*To the Editor of* The Hospital.

### A Plea for the Private Nursing Home.

*To the Editor of* The Hospital.

Sir,—Would you care for the views of one in the work for the past eight and a half years in reply to, and with every respect for, your criticisms in a recent number? The name that we use suggests something that ought to be, and is, very different from both a hospital and a hotel: that of a home given up to the service of the sick; so much more is implied in it than mere food and lodging, and nursing even, and given the head is a gentlewoman with the tastes and instincts and upbringing of the class of her patients, that she has undergone a thorough general and special training for the class of cases undertaken, that she has had experience of both home and institutional housekeeping, and that she has control of sufficient capital for needs, she should provide an ideal home for the sick. My experience is that invalids do not want a hotel, and that hotels are not suitable places to nurse in; and the very name of hospital with its students and discipline and rules is not only enough to frighten many delicately nurtured people more sensitive than usual because sick, but the institution is essentially unsuited to the wants of the upper classes and never was intended for them before the late new departure.

I do not believe in nursing homes being run by medical men or women; it is not their province to see into the minutiæ of a house and the nursing of patients, and that every detail must be scrutinised by the head is a *sine qua non* of successful management. But the running of such a private nursing home strikes me as being particularly suitable to the class of women described, perhaps bordering on middle age, with all the education and social status required and plenty of experience at their back, but possibly no longer able for the rush of continuous ward work, and with talents unused therein which they would like to put to some account, feeling the circumscribing influences of incessant routine and limited leisure, and longing for more association with equals in intellect and aims, and yet being obliged to provide for themselves, they have talents to use for their own growth and others' good. The gauche *faux pas* you indicate and rightly condemn would be almost impossible of occurrence under this kind of management; humbugs of all kinds, and incapable people from any one cause, could not run a successful home, and should not try to; but patients whose houses are far distant, unsuitable for any reason, or which cannot be given up to the sick, are exceedingly glad to avail themselves of a good nursing home; they are able to pay the full cost, and why should they not do so? A large institution must of necessity lack much that a home supplies, be it never so well run and so essential to its own work in hand; but it lacks use for the special talents we describe as required for the private nursing home, which should never be so large as to need much division of supervision; rather it should be under the influence of one personality, who may continue in it to near old age, with adequate assistance: profiting by experience, the character grows richer to the advantage of the home.

The like of this is impossible in any other branch of the profession; how many private nurses are required after fifty years of age? how many matrons, or staff nurses, or sisters? The private nursing home thus offers the best place of treatment for a large class of patients and the most suitable congenial and remunerative work for a large class of nurses. Often the want of sufficient capital is the great bar to success, but co-operation amongst nurses should remove this difficulty. Again, another reason for: the doctor is very largely dependent for his success with cases from the day he enters hospital as a student to the days of general practice or specialising on the work of nurses, which he obtains always without cost to himself; does he not owe to them morally his support and help in running their own nursing homes as a slight compensation? at the same time doing neither to himself or his patients any harm, but rather saving and helping both, saving his patient and himself from countless worries and anxieties, and helping by finding everything in order and at hand as required in a way that never is in a private house, so that his work is better accomplished and time saved? Hospital posts cannot be said to offer a living wage to the average nurse, and there are very few pensions; and besides pensions should be for the prematurely disabled; and such responsible and arduous work as nursing ought to command sufficient remuneration to provide for maintenance during life.—I am, Sir, yours, etc.,

M. E. B.

India.

